# The Ottawa SAH search algorithms: protocol for a multi- centre validation study of primary subarachnoid hemorrhage prediction models using health administrative data (the SAHepi prediction study protocol)

**DOI:** 10.1186/s12874-018-0553-3

**Published:** 2018-09-15

**Authors:** S. W. English, L. McIntyre, V. Saigle, M. Chassé, D. A. Fergusson, A. F. Turgeon, F. Lauzier, D. Griesdale, A. Garland, R. Zarychanski, A. Algird, C. van Walraven

**Affiliations:** 1 Department of Medicine (Critical Care), University of Ottawa, Ottawa, ON K1Y 4E9 Canada; 2 Clinical Epidemiology Program, Ottawa Hospital Research Institute, Ottawa, ON Canada; 3 Department of Medicine, Division of Critical Care, Centre Hospitalier de l’Université de Montréal, Montreal, QC Canada; 4 Centre de recherche du CHU de Québec, Population Health and Optimal Health Practices Research Unit (Trauma – Emergency – Critical Care Medicine), Université Laval, Québec City, QC Canada; 5 Department of Anesthesiology and Critical Care Medicine, Division of Critical Care Medicine, Université Laval, Québec City, QC Canada; 6 Centre de recherche du Centre Hospitalier de l’Université de Québec, Université Laval, Québec City, QC Canada; 7 Deparment of Anesthesiology, Pharmacology & Therapeutics, University of British Columbia, Vancouver, Canada; 8 Department of Internal Medicine, Sections of Critical Care and Respirology, University of Manitoba, Winnipeg, MB Canada; 9 Department of Internal Medicine, Sections of Critical Care and Hematology/Medical Oncology, University of Manitoba, Winnipeg, MB Canada; 10 Department of Neurosurgy, McMaster University, Hamilton Health Sciences, Hamilton, ON Canada; 11 Department of Medicine, University of Ottawa, Ottawa, Canada

**Keywords:** Administrative health data, Prediction rule, Diagnosis, Subarachnoid hemorrhage

## Abstract

**Background:**

Conducting prospective epidemiological studies of hospitalized patients with rare diseases like primary subarachnoid hemorrhage (pSAH) are difficult due to time and budgetary constraints. Routinely collected administrative data could remove these barriers. We derived and validated 3 algorithms to identify hospitalized patients with a high probability of pSAH using administrative data. We aim to externally validate their performance in four hospitals across Canada.

**Methods:**

Eligible patients include those ≥18 years of age admitted to these centres from January 1, 2012 to December 31, 2013. We will include patients whose discharge abstracts contain predictive variables identified in the models (ICD-10-CA diagnostic codes I60** (subarachnoid hemorrhage), I61** (intracranial hemorrhage), 162** (other nontrauma intracranial hemorrhage), I67** (other cerebrovascular disease), S06** (intracranial injury), G97 (other postprocedural nervous system disorder) and CCI procedural codes 1JW51 (occlusion of intracranial vessels), 1JE51 (carotid artery inclusion), 3JW10 (intracranial vessel imaging), 3FY20 (CT scan (soft tissue of neck)), and 3OT20 (CT scan (abdominal cavity)). The algorithms will be applied to each patient and the diagnosis confirmed via chart review. We will assess each model’s sensitivity, specificity, negative and positive predictive value across the sites.

**Discussion:**

Validating the Ottawa SAH Prediction Algorithms will provide a way to accurately identify large SAH cohorts, thereby furthering research and altering care.

## Background

In the presence of an *incomplete* disease cohort, the epidemiologic study of any disease can produce biased results. This is because large and complete cohorts of patients are needed to accurately understand population characteristics, prognostic factors, long-term outcomes and disease burden. Unfortunately, prospective studies of rare diseases are often not feasible due to the exorbitant cost and time required to identify and assemble sufficient cohorts. Consequently, researchers turn to retrospective study designs that lack completeness or accuracy and may lead to inaccurate estimates of disease burden, mortality, and healthcare resource utilization which, in turn, could prompt the development of inappropriate or potentially harmful strategies to care for people with these rare diseases.

One such rare disease is primary subarachnoid hemorrhage (pSAH), a devastating illness that is predominantly the result of a ruptured arterial aneurysm or arteriovenous malformation (AVM) [1–4]. Most affected patients are between the ages of 40 and 60 years [5, 6]. The incidence of SAH has varied between studies. A 2007 systematic review of prospective studies specifically examining SAH incidence demonstrated a range from 4.2 (95% CI 3.1 to 5.7) to 22.7 (95% CI 21.9 to 23.5) per 100,000 person-years [7]. Although this review included four North American studies, they all predated 1990. More recent American and Canadian studies (from the 1990s) suggest an incidence of 8.0 to 11.2/100,000 patient years [2, 8]. These studies were retrospective in design and used diagnostic codes for case-ascertainment. Case-fatality rates vary by region and by case-defining methods. For example, they range from 23 to 62% in studies where diagnostic codes were used for disease identification [2, 9–12].

A possible source of these widely variable results is the methods used to retrospectively create these cohorts. Few studies have conducted a detailed examination of the validity of using diagnostic codes to identify pSAH (see Table 1). Ellekjaer et al. concluded from their study using discharge data of stroke patients that diagnostic codes should not be used to identify the subtypes of stroke, including pSAH, because of incidence overestimation [13]. In a review by Williams et al., the positive predictive value (PPV) of diagnostic codes for pSAH was found to vary from 64 to 100%, with the higher values coming from the smaller studies (all under 30 patients) [8]. Mayo et al. [14] summarized 4 studies [14–17] that have examined the accuracy of diagnostic coding. Although the probability that a patient with the diagnostic code for pSAH (ICD-9-CM 430) actually had the disease (based on clinical review) ranged from 33 to 100%, the prevalence of pSAH in the populations examined ranged anywhere from 12.5 to < 1%. Further, the potential for missed cases was not accounted for and, thus, neither the specificity nor sensitivity can be calculated and only reported in two studies [18, 19].

**Table 1:** Summary of literature describing the accuracy of diagnostic codes for SAH

Study	Total sample size	Number with ICD code(s) for SAH	Proportion of those with code truly having SAH (PPV)	Diagnostic code sensitivity/specificity, % (95% CI)
Liu L et al. (1993) [15]	683	14	92.9%	Not examined
Phillips SJ et al. (1993) [16]	301	3	33%	Not examined
Mayo N et al. (1993) [17]	96	1	100%	Not examined
Mayo N et al. (1993) [17]	3197	247	94.7%	Not examined
Leibson CL et al. (1994) [27]	364	11	100%	Not examined
Broderick J et al. (1998) [28]	Not stated	14	64%	Not examined
Rosamond WD et el. (1999) [29]	1185	22	86%	Not examined
Roumie CL et al. (1998) [30]	231	2	100%	Not examined
Tirschwell et al. (2002) [18]	206	58	86%	Sens = 98 (90–100) Spec = 92 (84–96)
Kokotailo RA et al. (2005) [31]	461 (ICD-9)/256 (ICD-10)	51/32	98% (90–99)/91% (77–98)	Not examined
Jones SA et al. (2014) [19]	4260	56	79% (66–88)	Sens = 93 (92–99)

*CI* confidence interval, *NR* not reported, *PPV* positive predictive value, *Sens* sensitivity, *Spec* Specificity

There is great potential in using health administrative data to study pSAH if cases can be accurately identified. Health administrative data are routinely collected to create a description of most health care encounters, including hospital admissions, by summarizing diagnostic, procedural, demographic, and administrative information. These summaries, commonly known as discharge abstracts, are created for each patient hospitalization. The interventions and diagnoses the patient received during their course of stay are captured with a code (ICD or CCI, respectively). Using this health administrative data to identify complete pSAH disease cohorts would significantly reduce the time and effort needed to complete these sorts of epidemiological surveys.

We have previously derived and validated a prediction model to retrospectively identify all hospitalized patients with a high probability of having suffered a pSAH using administrative data at a single centre [20, 21]. We have since derived two other prediction models (publication pending). Our published model had a sensitivity of 96.5% (95% CI 93.9–98.0), a specificity of 99.8% (95% CI: 99.6–99.9%), a positive likelihood ratio (+LR) of 483 (95% CI: 254–876), and a positive predictive value of 96.8% (95% CI: 94.3–98.3%). Patients with a high likelihood of pSAH are identified by estimating the probability a pSAH occurred based on the presence or absence of a number of variables, including the ICD and CCI codes. Externally validating The Ottawa SAH Search Algorithms will determine whether they can be used to identify pSAH patients admitted to other hospitals, thereby permitting the proper study of this important disease and a better understanding of those affected by it, their prognosis, and long-term outcomes. Additionally, these algorithms could be used to investigate the type and amount of care administered to patients with pSAH during their hospital stay. Here, we propose the methods of externally validating these prediction models to assess their generalizability to justify their further use.

## Methods

### Aim

We aim to test the accuracy of The Ottawa SAH Search Algorithms in patients ≥18 years of age admitted to four Canadian tertiary care centres between January 1, 2012 and December 31, 2013 (Vancouver General Hospital, Winnipeg Health Sciences Centre, Hamilton Health Sciences Centre, and Hôpital de L’Enfant-Jésus du CHU de Québec-Université Laval).

### Objectives

#### Primary

To describe the performance metrics (sensitivity, specificity, positive and negative prediction values, and likelihood ratios) of The Ottawa SAH Search Algorithms using routinely collected health administrative data from 4 Canadian academic tertiary care health centres.

#### Secondary

- To identify and describe differences in performance characteristics of the pSAH prediction models between institutions.
- To identify and describe differences in performance characteristics of the pSAH prediction models using varying predicted probability thresholds (e.g., thresholds of 50, 75, 90%).
- To create a complete 2-year multi-centre cohort of hospitalized patients with pSAH.

### Study hypothesis

We aim to prove our hypothesis that The Ottawa SAH Search Algorithms will identify all patients with pSAH at 4 separate Canadian Academic Hospitals with at least 95% sensitivity (within a 2.5% margin of error).

### Study population

The population of interest will include patients ≥18 years of age who were admitted to one of the study hospitals between January 1, 2012 to December 31, 2013 and whose discharge abstract contain specific variable values (detailed below).

### The Ottawa SAH search algorithms

We will test each of 3 algorithms:

#### Recursive partitioning model

This original algorithm has been previously published and described in detail elsewhere [21]. Essentially this algorithm was created using recursive partitioning to identify the combination of diagnostic and procedural codes, as well as other health administrative data, that most accurately identified SAH. The model was validated on a separate data set (Table 2 A).

**Table 2:** Ottawa SAH prediction algorithm pathways and anticipated predictive probability of each pathway based on our experience at The Ottawa Hospital

Prediction Model A – Recursive Partitioning Model													
Prediction Model Pathways	Diagnostic codes (ICD10CA)					Procedural codes (CCI)			Admission characteristics			Predicted probability of pSAH (%)	
	SAH (ICD 160)	ICH (ICD 161)	Other nontraumatic ICH (ICD 162)	Other CVD (ICD 167)	Intracranial Injury (ICD S06)	Therapeutic Carotid Artery Occlusion (CCI 1JE51)	Therapeutic Occlusion Intracranial Vessels (CCI 1JW51)	Extracranial Vessel Imaging (CCI 3JW10)	LOS (days)	Admission Type			
1	+											97.6	
2	+						+					100	
3	+						–		≥2			83.7	
4	+						–		< 2			100	
5	–						+					76.9	
6	–	+					–					41.2	
7	–	+					–			Urgent		28.6	
8	–	+					–			Nonurgent		100	
9	–	–				+	–					100	
10	–	–		+		–	–					26.7	
11	–	–		+		–	–	+				100	
12	–	–		+		–	–	–				15.4	
13	–	–	+	–			–					28.6	
14	–	–	–	–	+		–					10.3	
15	–	–	–	–	+		–			Urgent		2.9	
16	–	–	–	–	+		–			Nonurgent		75	
17	–	–	–	–	–		–					0.1	
Prediction Model B – SAH prediction point system													
Prediction Variables	Diagnostic codes (ICD10CA)					Procedural codes (CCI)				Admission characteristics			
	SAH (ICD 160)	ICH (ICD 161)	Other CVD (ICD 167)	Intracranial Injury (ICD S06)	Other postprocedural nervous system disorder (ICD G97)	Therapeutic Occlusion Intracranial Vessels (CCI 1JW51)	Extracranial Vessel Imaging (CCI 3JW10)	CT scan (soft tissue of neck) (3FY20)	CT scan (abdominal cavity) (3OT20)	LOS		Admission Type (Urgent or Emergent)	Admitted to ICU
										≤ 2 days	≥ 3 days		
Points	6	4	4	3	4	3	1	−3	−2	0	−1	2	1
Score									Predicted probability of pSAH (%)				
≤ 0									0				
1									0.1				
2									0.1				
3									1.4				
4									2.4				
5									17.2				
6									33.3				
7									63.9				
8									94.6				
9									97.0				
10									100				
11									100				
12									98.8				
≥ 13									100				
Prediction Model C – Prevalence-adjusted SAH Prediction Point System													
Prediction Variables	Diagnostic codes (ICD10CA)							Procedural codes (CCI)					
	SAH (ICD 160)				ICH (ICD 161)			Therapeutic Occlusion Intracranial Vessels (CCI 1JW51)					
Points	2				1			1					
Score									50%tile Predicted probability of pSAH (%) (2.5–97.5%ile)				
0									0 (0–0.01)				
1									2.5 (0–5.6)				
2									20 (5–35.3)				
3									100 (100–100)				
4									100 (100–100)				

Cells containing “+” indicate that the relevant code was present and of interest to the pathway, “-” indicates that the code was of interest to the pathway, but was absent. Blank cells indicate that the corresponding code was not considered for that pathway. *ICD10CA* International Statistical Classification of Diseases and Related Health Problems, 10th Revision, Canada, *ICD* International Classification of Diseases and Related Health Problems, *CCI* Canadian Classification of Health Interventions, *SAH* subarachnoid hemorrhage, *ICH* intracranial hemorrhage, *CVD* cerebrovascular disease, *LOS* length of stay, *pSAH* primary subarachnoid hemorrhage

#### SAH prediction point system

For this model, the entire dataset from A (both derivation and validation data sets) were combined to generate the SAH prediction point system. This was done using logistic regression. We initially included all covariates with a univariable association with SAH *p*-value of < 0.2, and then removed those that did not have a *p*-value < 0.05 during subsequent backward variable selection. The final logistic regression model was converted into a point system [22]. Each significant variable was assigned a point value ranging from − 3 to 6 based on the likelihood that the presence of this variable in discharge abstracts was associated with pSAH. Positive points indicate that patients with these variables were more likely to have pSAH, whereas negative points indicate variables rarely seen in the discharge abstracts of patients with pSAH. The sum of these points is tied to each patient’s overall predictive probability of having pSAH (Table 2 B).

#### Prevalence-adjusted SAH prediction point system

For this model, we repeated the methods in B except we used bootstrap methods described by Austin et al. for variable selection [23]. This method is more restrictive and resulted in a more parsimonious model. These methods usually help avoid spurious variables to be included in the model. The final model was converted into a point system again [22]. To more accurately predict the probability of SAS with each possible point score, we used a baseline actual SAH hospital prevalence of 16.7 per 10,000 admissions [20]. To do this, we created bootstrap samples stratified by SAH status using ratios between cases and controls that resulted in SAH prevalence within each bootstrap sample that ranged randomly around 16.7 per 10,000. Within each bootstrap sample (with each having an overall SAH prevalence similar to that which would be expected in teaching hospitals similar to ours) we measured the probability of SAH in each SAH point score. The final expected SAH probability for each point score was the median of the 500 bootstrap samples (Table 2 C).

We are testing the three algorithms to identify which works best across multiple jurisdictions and health systems, as in the sites included in our sample.

### Study procedures

**Fig. 1:**
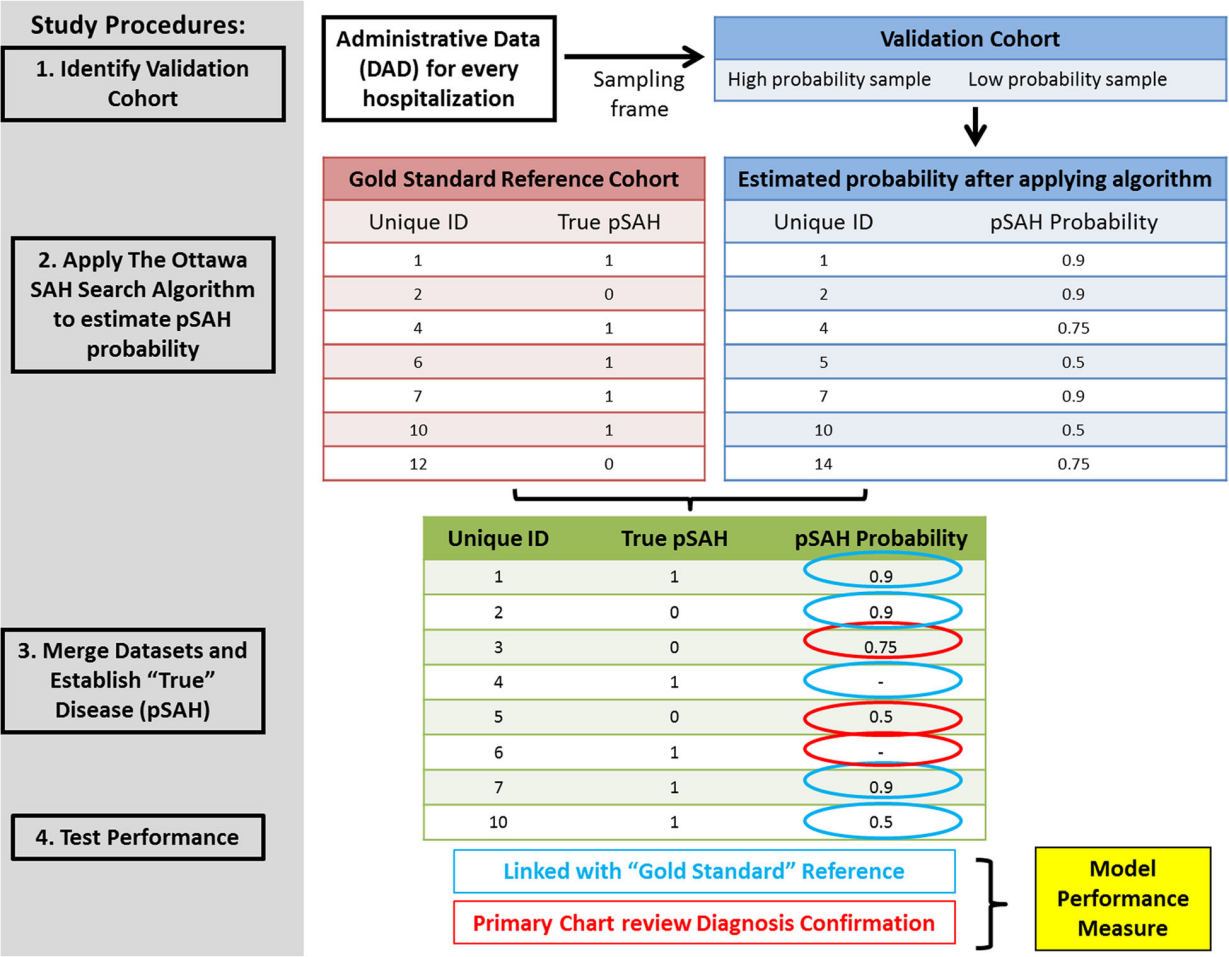
Proposed Study Methods implementing a TOH SAH search algorithm

The study objectives will be achieved by following four main steps (Fig. 1):

1. Identifying the validation cohort
2. Applying The Ottawa SAH Search Algorithms
3. Establishing “true” disease (pSAH) via chart review
4. Testing The Ottawa SAH Search Algorithms performance

#### Step 1: Identifying validation cohort

We will identify all patients in the validation cohort using administrative health data from each site. From every hospitalization over the study period, we will create a sampling frame comprised of a “high probability” and “low probability” sample. We define pSAH as a subarachnoid hemorrhage (identified on CT head scan, lumbar puncture or autopsy) that was the result of a ruptured aneurysm or AVM (identified on angiography or autopsy) [20].

##### High probability sample

The high probability sample will be composed of all patients whose discharge abstracts contain at least one of the diagnostic or procedural predictor variables contained in the prediction model and that correspond to the prediction model pathways outlined in Table 2.

##### Low probability sample

This sample will consist of a random sample of unique patient admissions (max. *N* = 300 per site) who are anticipated to have a very low predicted probability of having pSAH based on The Ottawa SAH Search Algorithms. That is to say, patients who either do not have any of the diagnostic or procedural pSAH predictor variables (see pathway 17 in Table 2) or that have one or more of the variables but who do not otherwise meet one of the prediction model pathways. Excluded from this sample will be all admissions to psychiatry or obstetrics as these admissions are extremely unlikely to be related to pSAH and and, thus, could erroneously augment the negative predictive value of the algorithm. Across 4 centres, we will ensure a minimum of 1200 ‘predicted pSAH negative’ group to allow adequate power for the calculation of external specificity and sensitivity of the model.

For each entry in the validation group, we will abstract the following: admission and discharge date; age at admission; sex; type of admission (emergency, nonurgent, unknown); length of stay; whether they were admitted to the ICU; and the presence or absence of ICD-10-CA diagnostic codes I60** (subarachnoid hemorrhage), I61** (intracranial hemorrhage), 162** (other nontrauma intracranial hemorrhage), I67** (other cerebrovascular disease), S06** (intracranial injury), G97 (other postprocedural nervous system disorder) and CCI procedural codes 1JW51 (occlusion of intracranial vessels), 1JE51 (carotid artery inclusion), 3JW10 (intracranial vessel imaging), 3FY20 (CT scan (soft tissue of neck)), and 3OT20 (CT scan (abdominal cavity)). An anonymized unique study identifier will be assigned to each entry and tracked in a master log kept at each respective study centre.

#### Step 2: Applying the Ottawa SAH search algorithms

At each study centre, all patient identifiers will be removed from the study dataset and replaced with a de-identified study identification. These datasets will be encrypted and sent to the host centre to be merged into one large validation set, where it will be password-protected and stored on the hospital server per hospital policy. We will apply The Ottawa SAH Search Algorithms to the entire cohort to assign a predicted probability of pSAH to each patient.

#### Step 3: Establishing “true” disease (pSAH) status

The ‘true’ disease status will be determined in all screen positive patients (i.e. those who have a high predicted probability of having pSAH) and the low probability sample (i.e. 1200 screen negative patients). ‘True’ disease status will be established by: a) linking to a gold standard reference; *or* b) primary diagnosis verification (see Fig. 1).

#### Linking to a “gold standard” reference cohort

Established pSAH cohorts exist at each of the study centres during the time period under investigation. Three centres previously participated in a cohort study of patients with primary SAH [24] (Vancouver, Winnipeg, Quebec City) and one has a pre-existing pSAH registry (Hamilton). All admissions in these datasets have already been confirmed to have pSAH, based on the pSAH definition outlined in Step 1. We will link patients from the study sample dataset to the “Gold Standard” reference. Therefore, we will know the true disease status of all patients for which a link is made. Any patient with true pSAH in the “Gold Standard” reference cohort over the study period for which there is no link in the validation cohort will be considered a patient missed by the algorithm and count as a false negative. Any entry in the validation cohort that is not linked with an entry in the “Gold Standard” reference cohort will undergo primary chart review verification (see b in Fig. 1).

#### Primary diagnosis verification (chart review)

All patients for which no linkage occurred in (a) and/or for whom the true pSAH status is unknown will undergo a medical record review to ascertain their true pSAH status.

#### Step 4: Testing algorithm performance (analysis)

Algorithm performance will be measured by generating 2 × 2 tables to determine sensitivity, specificity, and predictive values when testing the expected outcome (see Table 2) compared with the observed outcome. Three classifications for determining expected outcomes will be tested: one in which the predicted probability of having pSAH is ≥50%, ≥75% and, finally, a more specific criteria of an observed event rate ≥ 90%. Model performance (accuracy) will be measured by comparing expected and observed number of patients with pSAH with 2 × 2 tables to calculate sensitivity, specificity and predictive values with 95% confidence intervals. SAS 9.3 (North Carolina USA, OHRI license) will be used to implement the algorithm and run all analyses. We will assess performance across all 4 centres collectively and individually.

### Sample size

To ensure our algorithms have a sensitivity of 95% with a margin of error ≤ 2.5%, a minimum sample of 292 patients with pSAH is required. Based on the previous work [24, 25], we expect that over 500 patients from the 4 study centres will have had an admission for pSAH during the study period. Assuming a Poisson distribution, a minimum of 1000 screen negative cases will need to be reviewed to ensure that the true false negative proportion of our models have an upper 95% confidence limit of ≤5%. The proposed study design accomplishes: 1) the necessary sample size to meet our goals; 2) multiple years to demonstrate consistency of the prediction model over time; and 3) multiple sites to demonstrate generalizability across the vast Canadian geography and different health care models between provinces.

### Ethics and consent

Approval from each site’s local research ethics boards and that of the sponsoring institution will be maintained throughout the duration of the study. Since no direct patient contact is required given the retrospective nature of this study, a waived consent model will be implemented.

## Proposed timelines

We estimate a study duration of 16 months from the time of host centre REB approval (granted in December 2016). Participating center identification is complete and funding has been attained (Ontario Ministry of Health Innovation Grant through The Ottawa Hospital Academic Medical Organization). We intend to complete analysis by October 2018.

## Discussion

For rare diseases such as pSAH, retrospective identification of patient cohorts using single diagnostic codes is problematic when their accuracy is based predominantly on their positive predictive values. Since accuracy of positive predictive value is affected by disease prevalence [26] the reliability of the case ascertainment may be limited. Given the limited available accuracy measures [14–18, 27–29], it is highly plausible that any admitted patient with an ICD code for SAH has only a 1 in 100 chance of actually having the diagnosis [20, 21]. More accurate means to reliably identify pSAH retrospectively are necessary. We intend to test if one or all of the Ottawa SAH Prediction Algorithms could serve this purpose.

### Project influence on the healthcare system and patient care

To accurately study a disease and understand its prognosis and outcomes, complete and accurate groups of patients with that disease must be identified. Given the substantial challenges of accurately and effectively doing this with rare diseases like pSAH, researchers have turned to questionably reliable methods of creating such groups for study. Externally validating The Ottawa SAH Search Algorithms, as proposed in this study, will provide researchers (and, in turn, knowledge users) with a reliable, easy and cost-effective means of accurately identifying groups of hospitalized patients with pSAH to further our understanding of this disease process, its prognosis, and its outcomes. Such a study will not only influence patient care and the healthcare system approach to this important population, but will also influence our choice of intervention and its measure of effect. This study has the potential to not only directly impact the researcher and his/her plight to understand and intervene on this important disease, but also on the frontline healthcare provider whose understanding, prognostic decisions and interventions are predicated on the fundamental understanding of pSAH.

## Abbreviations

AVM: Arteriovenous malformation
CCI: Canadian Classification of Health Interventions
ICD10CA: International Statistical Classification of Diseases and Related Health Problems, 10th Revision, Canada
pSAH: primary subarachnoid hemorrhage
SAH: Subarachnoid hemorrhage
